# Periconceptional GLP-1 receptor agonist exposure and obstetric outcomes: a Danish nationwide cohort study

**DOI:** 10.1093/hropen/hoag015

**Published:** 2026-03-18

**Authors:** Kathrine Vauvert R Hviid, Karina Banasik, Laust Hvas Mortensen, Sten Madsbad, Katrine Strandberg-Larsen, Nina Rica Wium Geiker, David Westergaard, Henriette Svarre Nielsen

**Affiliations:** Department of Gynecology and Obstetrics, Copenhagen University Hospital Hvidovre, Hvidovre, Denmark; Institute of Clinical Medicine, Faculty of Health and Medical Sciences, University of Copenhagen, Copenhagen, Denmark; Department of Gynecology and Obstetrics, Copenhagen University Hospital Hvidovre, Hvidovre, Denmark; Department of Health Technology, Technical University of Denmark, Lyngby, Denmark; Methods and Analysis, Statistics Denmark, Copenhagen, Denmark; Department of Public Health, University of Copenhagen, Copenhagen, Denmark; ROCKWOOL Foundation Research Unit, Copenhagen, Denmark; Institute of Clinical Medicine, Faculty of Health and Medical Sciences, University of Copenhagen, Copenhagen, Denmark; Department of Endocrinology, Copenhagen University Hospital Hvidovre, Hvidovre, Denmark; Department of Public Health, University of Copenhagen, Copenhagen, Denmark; Centre for Childhood Health, Copenhagen, Denmark; Department of Nutrition, Exercise and Sports, University of Copenhagen, Copenhagen, Denmark; Department of Gynecology and Obstetrics, Copenhagen University Hospital Hvidovre, Hvidovre, Denmark; Department of Health Technology, Technical University of Denmark, Lyngby, Denmark; Methods and Analysis, Statistics Denmark, Copenhagen, Denmark; Department of Gynecology and Obstetrics, Copenhagen University Hospital Hvidovre, Hvidovre, Denmark; Institute of Clinical Medicine, Faculty of Health and Medical Sciences, University of Copenhagen, Copenhagen, Denmark

**Keywords:** GLP-1 receptor agonist, pregnancy, preterm birth, diabetes, obesity, periconceptional exposure, liraglutide, semaglutide

## Abstract

**STUDY QUESTION:**

What is the association between periconceptional GLP-1 receptor agonist exposure and risk of obstetric complications?

**SUMMARY ANSWER:**

Periconceptional GLP-1 receptor agonist exposure was associated with increased preterm birth risk when used for diabetes treatment (liraglutide aOR 1.70, 95% CI 1.17–2.48; semaglutide aOR 1.84, 95% CI 1.24–2.7) but not for weight management, suggesting the underlying diabetes rather than the medication may be the causal factor.

**WHAT IS KNOWN ALREADY:**

GLP-1 receptor agonists are rapidly expanding in use among reproductive-age women for diabetes and obesity treatment. While not approved for use in pregnancy, inadvertent periconceptional exposure occurs frequently. Limited safety data exist, with recent small studies suggesting no increased risk of major congenital malformations, but comprehensive obstetric outcome data remain lacking.

**STUDY DESIGN, SIZE, DURATION:**

This nationwide observational cohort study used data from Danish health registries from October 2009 through December 2023. We analyzed 756 636 singleton pregnancies among 480 231 women, with 529 pregnancies having periconceptional GLP-1 receptor agonist exposure.

**PARTICIPANTS/MATERIALS, SETTING, METHODS:**

We identified periconceptional liraglutide or semaglutide exposure (prescription redemption within 8 weeks before/after last menstrual period) using the National Prescription Register. Exposed pregnancies were stratified by maternal pre-existing diabetes status and compared with propensity score-matched unexposed controls. Propensity score matching incorporated maternal age, BMI, smoking, geographic region, education, pre-existing diabetes, parity, and temporal factors.

**MAIN RESULTS AND THE ROLE OF CHANCE:**

Before adjustment, exposed women had higher rates of multiple obstetric complications. After propensity score matching, only the risk of preterm birth remained elevated for exposed women. This increased risk was confined to women using GLP-1 receptor agonists for diabetes treatment (liraglutide aOR 1.70, 95% CI 1.17–2.48; semaglutide aOR 1.84, 95% CI 1.24–2.71). Among women without pre-existing diabetes using these medications for weight management, no association with preterm birth was observed (liraglutide aOR 1.01, 95% CI 0.58–1.76; semaglutide aOR 0.71, 95% CI 0.30–1.70).

**LIMITATIONS, REASONS FOR CAUTION:**

Study limitations include the absence of data regarding medication compliance post prescription redemption, potential misclassification due to parallel importation, and the inability to control for unmeasured confounding factors. The observational design cannot establish causality. Most semaglutide weight-loss prescriptions occurred late in the study period, limiting long-term follow-up data.

**WIDER IMPLICATIONS OF THE FINDINGS:**

The results are compatible with the hypothesis that diabetes-related factors, rather than GLP-1 receptor agonist exposure itself, may contribute to the increased preterm birth risk. If so, there are important implications for preconception counselling and may inform future guidelines for GLP-1 receptor agonist use in reproductive-age women.

**STUDY FUNDING/COMPETING INTEREST(S):**

KVRH, KB, DW, and HSN acknowledge funding from the Novo Nordisk Foundation (NNF21OC0069257 and NNF220C0077221) and the AP Moller Foundation. LMH was supported in part by grants from NordForsk (id: 105545), the Novo Nordisk Foundation (NNF17OC0027594 and NNF17OC0027812), and the Villum Foundation (‘Nation-Scale Social Networks’). KSL was supported by the Independent Research Fund Denmark (8045-00047B), NordForsk (id: 156298), and Centre for Childhood Health (id: 72 2024_F_008 and 2024_I_001). SM: Advisory boards: AstraZeneca, Boehringer Ingelheim, Intarcia Therapeutics, Novo Nordisk, Sanofi, Abbott Lab, Bayer, Amgen; Lecture fees: AstraZeneca, Novo Nordisk, MSD; Research grant recipient: Novo Nordisk; Novo Nordisk foundation, Boehringer Ingelheim; Support for attending meetings and/or travel: Novo Nordisk, Boehringer-Ingelheim, Bayer. Grants were paid to the institution, Hvidovre Hospital, University of Copenhagen, with no personal fee. None of the grants has any relation to the work presented in the paper. SM is also a consultant for Netdoktor and has served as principal investigator in relation to the development of drugs for the treatment of type 2 diabetes and obesity in collaboration with Novo Nordisk and Bayer, with funds paid to the institution where he is employed, with no personal fee and with no relation to the work reported in this article. HSN: lecture fees on own research: Novo Nordisk A/S, Ferring Pharmaceuticals, Merck A/S, Astra Zeneca, Cook Medical, Gedeon Richter and Ibsa Nordic. K.B holds stock/share or stock/share options with Novonesis, Genmab, and Novo Nordisk (held in personal investment portfolio). The remaining authors have no other disclosures.

**TRIAL REGISTRATION NUMBER:**

N/A

WHAT DOES THIS MEAN FOR PATIENTS? This large-scale Danish study examined pregnancy outcomes in women inadvertently exposed to GLP-1 receptor agonists during early pregnancy. Among over 750 000 pregnancies, researchers found that these medications were associated with increased preterm birth risk, but this risk was only present when the medication was used for diabetes treatment, not when used for weight management. This suggests that diabetes itself, rather than the medication, may be responsible for the increased risk. Although we are lacking data on how many women were actually using the medication after redeeming their prescriptions in the preconceptual period, the results may indicate that women without diabetes who become pregnant while using these increasingly popular weight-loss medications have no increased risk of complications or preterm birth.

## Introduction

The prevalence of overweight and obesity continues to increase globally, now affecting more than half the population in the European Region (World Health Organization, 2022; World Obesity Federation, 2022). In the forecast analysis of obesity trajectories published in *The Lancet* in March 2025, it is predicted that by 2050, up to 2 in 3 adults over 25 years will be overweight or obese (Ng *et al.*, 2025). In Denmark, 40–48% of women in the fertile age are overweight or obese, which increases their risk of obstetric and neonatal complications such as pre-eclampsia, gestational diabetes mellitus, preterm birth and giving birth to a child large-for-gestational-age (Marchi *et al.*, 2015; Poston *et al.*, 2016; Sundhedsstyrelsen *et al.*, 2022). Overweight and obesity are associated with increased risk of infertility including an increased time to pregnancy, ovulatory dysfunction, and when using assisted reproductive technology, a lower chance of pregnancy success (Van Der Steeg *et al.*, 2008; Wise *et al.*, 2010; Poston *et al.*, 2016). Weight reduction increases the chance of natural conception and reduces the risk of obstetric complications, leading to a particular interest in effective weight loss methods such as the glucagon-like peptide-1 receptor agonist (GLP-1 RA) among some women planning pregnancy (Best *et al.*, 2017; Price *et al.*, 2020; Hunter *et al.*, 2021; Schenkelaars *et al.*, 2021). The first generation of GLP-1 RAs typically induces a 5–10% weight loss, whereas the newest more potent generation, semaglutide 2.4 mg once weekly, has demonstrated a 15–16% weight loss (Wadden *et al.*, 2021; Wilding *et al.*, 2021; Garvey *et al.*, 2022; Weghuber *et al.*, 2022).

GLP-1 RAs are not approved for use during pregnancy, and contraception is strongly recommended during treatment and for a minimum of two months after discontinuation to avoid fetal toxicity (European Medicines Agency, 2019; European Medicines Agency, 2022a; European Medicines Agency, 2022b; European Medicines Agency, 2024). While safety data remains limited, recent studies on inadvertent exposure to GLP-1 RAs have appeared. Six observational studies have evaluated pregnancy outcomes after exposure to GLP-1 receptor agonists (GLP-1 RAs). Four studies have examined risks of major congenital malformations, all-cause maternal mortality and fetal cardiac or kidney anomalies, and none have found an increased risk after GLP-1 RA exposure compared with various reference groups. Cesta *et al.*, Dao *et al.*, and Hanif *et al.* included patients with type 2 diabetes, and compared women using GLP-1 RAs to either those using insulin treatment during the periconceptional period (Cesta *et al.*, 2024) or patients receiving non-GLP-1 RA antidiabetic drugs (mostly metformin) or women with overweight/obesity without diabetes (Dao *et al.*, 2024) or an unexposed control group, not further described (Hanif *et al.*, 2025). One study extracted data from the US Food and Drug Administration (FDA) on inadvertent exposure in women participating in clinical trials of GLP-1 Ras (Parker *et al.*, 2025). Two large retrospective cohort studies examined broader obstetric outcomes after GLP-1 RA use. In a single academic health system, Maya *et al.* (2025) reported that preconception or early-pregnancy GLP-1 RA exposure was associated with greater gestational weight gain and higher risks of preterm delivery, gestational diabetes and hypertensive disorders of pregnancy. In contrast, using the US TriNetX database, Imbroane *et al.* (2025) found that GLP-1 RA prescriptions within 24 months before pregnancy were associated with lower odds of gestational diabetes, hypertensive disorders of pregnancy, preterm delivery, and caesarean delivery. Case reports with a variant exposure time of GLP-1 RA during pregnancy report uneventful pregnancies and births, while animal model organism studies have shown signs of repro-toxicity with a reduction in embryonic survival, growth restriction, and skeletal malformation (Greco, 2015; Ivanišević *et al.*, 2018; European Medicines Agency, 2019; European Medicines Agency, 2022a; European Medicines Agency, 2022b; Muller *et al.*, 2023; Skov *et al.*, 2023; Abushanab *et al.*, 2025). A dosage-response effect must also be considered, but such studies have been very limited. The repro-toxicity found in the animal studies responds to a dose equal to the maximum human dose or higher than that used for the studied animals (Food and Drug Administration (FDA), 2017, 2023; Younes *et al.*, 2020). However, it remains unclear whether the cause is the drug itself or the decrease in intake of food and subsequent weight loss in the mother mice (Muller *et al.*, 2023; Drummond *et al.*, 2025).

Liraglutide and semaglutide, two types of GLP-1 RAs, resemble the native human GLP-1 hormone in their structure by 94–97% and have a molecular weight of 3751.3 Da and 4113.6 Da, for liraglutide and semaglutide, respectively (PDB-101: Protein Data Bank, 2025a; PDB-101: Protein Data Bank, 2025b). The possibility of a drug to transfer across the placenta is typically set below a molecular weight of 600 Da (Thomas and Yates, 2012). The high molecular weight of both liraglutide and semaglutide would theoretically prevent them from crossing placenta; however, to our knowledge, no studies have examined this. The newest systematic review by Muller *et al.* found only case reports with no evidence of transfer of liraglutide from the mother to child (Ivanišević *et al.*, 2018; Muller *et al.*, 2023). Additionally, only a few mouse model studies of exendin-4, another GLP-1 RA, were identified in the review to examine the possibility of transfer across the placenta to the offspring. With a molecular weight of 4186 Da, exendin-4 should not either be able to cross the placenta, however, when the pregnant mice had systemic inflammation, exendin-4 did cross the placenta (Garcia-Flores *et al.*, 2018; Muller *et al.*, 2023). Several reviews concur that the current recommendations remain based on precautionary principles and animal data, emphasizing the urgent need for robust human evidence regarding the effects of periconceptional GLP-1 RA exposure (Kuitunen, 2024; Maslin *et al.*, 2024; Drummond *et al.*, 2025; Finkle and Brost, 2025; Price and Nankervis, 2025).

Here, we conducted a nationwide observational study in 756 636 pregnancies in Denmark examining the association between inadvertent periconceptional GLP-1 RA exposure on obstetric outcomes, including pre-eclampsia, gestational diabetes mellitus (GDM), preterm birth, birth weight, placenta weight, stillbirth, placental abruption, and placenta previa.

## Materials and methods

### Permissions

The study was carried out according to the General Data Protection Regulation. According to Danish legislation, Medical Research Ethics Committee approval is not required for register-based studies, i.e. secondary analysis of administrative data. This study is registered with the Data Protection Agency via Statistics Denmark.

### Study design

**Figure 1.**
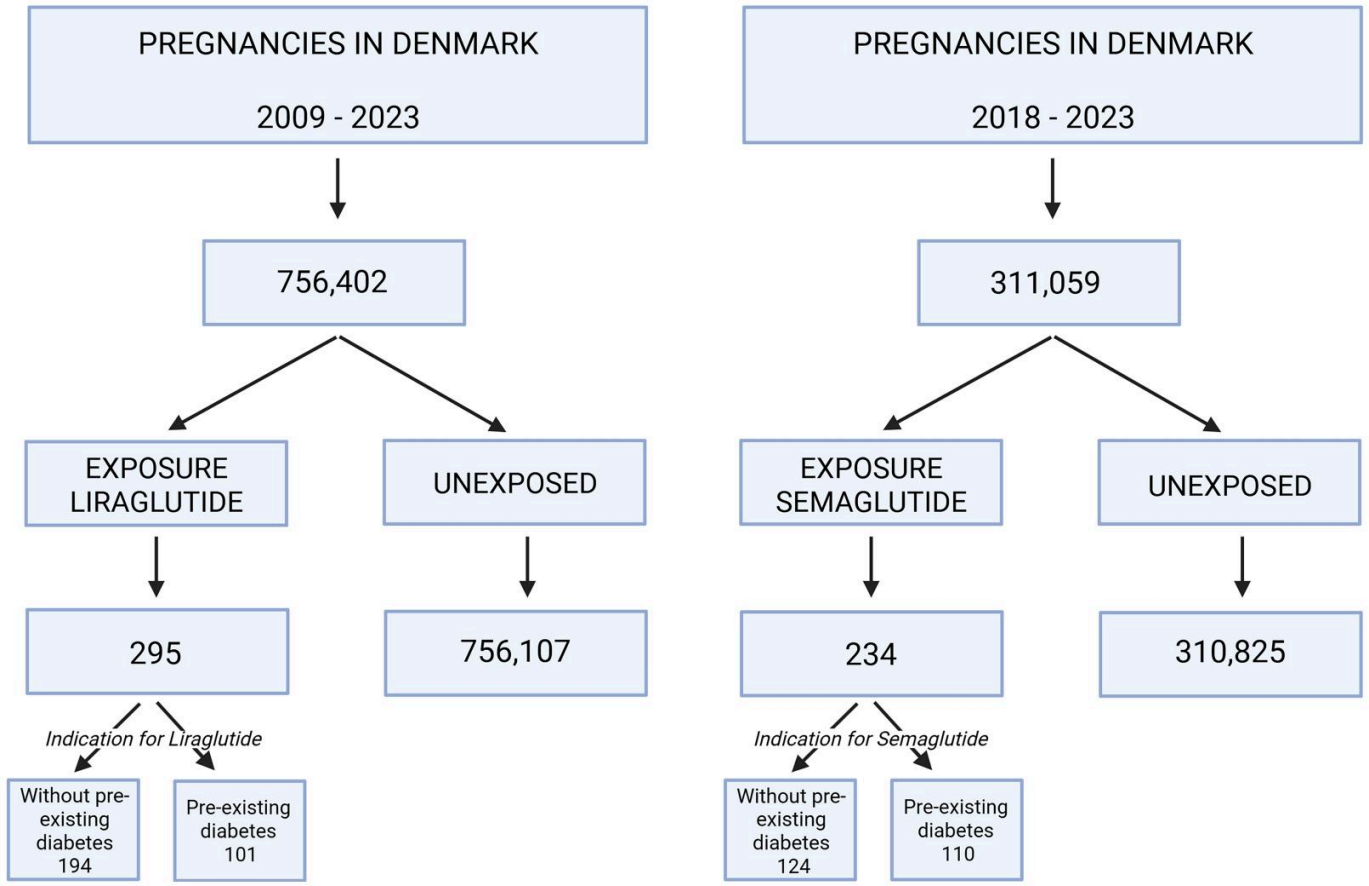
**Flowchart of inclusion into the study.** All pregnancies, with the exception of multifold pregnancies, in the time period 2009–2023 are identified from PREGDK, the unified pregnancy registry at Statistics Denmark. Pregnancies were categorized as exposed or unexposed based on prescription redemptions. A total of 756 636 pregnancies were included in the study between 2009–2023. Of which, 756,402 pregnancies were unexposed pregnancies, 295 pregnancies exposed to liraglutide and 234 pregnancies exposed to semaglutide.

This nationwide cohort study used Danish health registries. A unique personal identification number used in Denmark enables direct linkage of individual-level information across registries. We analyzed 756 636 singleton pregnancies among 480 231 women resulting in delivery from 1st October 2009 through 31st December 2023 (Fig. 1). The total of 756 636 pregnancies included the 529 pregnancies exposed to a GLP-1 RA. Data sources included the National Prescription Register (medication exposures), Medical Birth Register (pregnancy outcomes), National Patient Register (diagnoses), Educational Register (socioeconomic status), and Central Persons Register (demographics). All registries had complete information up until 31st December 2023.

### Exposure assessment

Periconceptional GLP-1 RA exposure was defined as liraglutide (ATC: A10BJ02) or semaglutide (ATC: A10BJ06) prescription redemption within ±8 weeks from the date of the last menstruation, calculated using the date of birth and gestational age of the child recorded in the medical birth registry (Supplementary Fig. S1). This 16-week window captures the period when pregnancy may be unrecognized and encompasses early organogenesis. Prescription redemptions are mandatorily recorded, providing complete capture. The Danish guidelines suggest a dosage of 1.8 mg daily in women with diabetes or 3.0 mg daily in women with obesity for liraglutide, and 1.0 mg once weekly for semaglutide for treatment of patients with type 2 diabetes or 2.4 mg once weekly for patients with obesity (Dansk Endokrinologisk Selskab, 2023).

### Covariates

Potential confounders were selected based on clinical knowledge. Demographic factors included maternal age (continuous), parity (0, 1, 2, 3+), and geographic region of residence at the time of conception (according to the calculated date of the last menstrual period). Socioeconomic factors included education level (DISCED-15 classification collapsed to: no formal/primary, secondary, undergraduate, postgraduate). Clinical factors included pre-pregnancy BMI (continuous), smoking status, and pre-existing diabetes. Temporal factors included year and month of pregnancy to control for secular trends and seasonality. Pre-existing diabetes was determined using a validated algorithm (Sundhedsdatabanken, 2025).

### Outcomes

Obstetric outcomes were pre-eclampsia, GDM, gestational age (days), birth weight (grams), placental weight (grams), stillbirth, placental abruption, and placenta previa. Obstetric complications were identified by ICD-10 codes (Supplementary Table S1) or birth records. Pre-term delivery was defined as delivery before the 37th completed gestational week (<37 + 0). Small for gestational age (SGA) was defined as being in the lowest 10th percentile when compared within groups of the same calendar year, the same sex, and the same completed week of gestation; while large for gestational age (LGA) was defined as being above the 90th percentile. Gestational age- and sex-specific percentiles for SGA/LGA were calculated within each birth year to account for secular trends, based on birth data, as intrauterine growth curves were not available.

### Statistical analysis

We used propensity score matching to create balanced comparison groups and estimate causal effects while controlling for measured confounders (see previous section). We constructed six unexposed control groups by propensity score matching on: (1) all confounders and (2) all confounders except BMI and pre-existing diabetes to assess the influence of the primary indications for treatment. For control groups 3–6, we matched on all confounders but multiplied pre-exposure BMIs by fixed percentages to create counterfactual scenarios testing what would have happened if unexposed women had higher baseline BMIs. This approach addresses the fact that BMI in the exposed group is potentially already affected by GLP-1 RA. Since the average causal effect of GLP-1 RAs on BMI is unknown, we constructed four control groups with pre-exposure BMIs that were 5% (3), 7.5% (4), 10% (5), and 15% (6) higher than the corresponding exposed group values.

For the propensity score matching, we utilized fast generalized full matching, which outputs a set of matching weights that can be used to balance controls (Ho *et al.*, 2011; Sävje *et al.*, 2021). The underlying distance for the fast generalized full matching model was calculated using a generalized additive model, with a probit link function, and a thin plate spline for BMI.

We assessed the matching based on the standardized mean difference and variance ratio, including polynomials up to degree two (for continuous variables), and all two-way interactions (Supplementary File S1). An absolute standardized mean difference < 10% and a variance ratio < 2 indicated a successful match. An iterative process was used, where thin plate regression splines and/or interactions between variables were added until balance was achieved. Love plots from the matching are shown in Supplementary Figs S2–S13.

We also attempted to utilize nearest neighbor matching, without replacement, but we could not reach a satisfactory match according to our criteria.

Continuous variables were summarized as mean (standard deviation), and categorical variables were summarized as frequency (percentage). Group comparisons used independent-sample *t*-tests (continuous variables) and Chi-squared tests (categorical variables). A two-tailed *P*-value below 0.05 was considered statistically significant.

Outcomes were compared using linear regression (for birth weight, placenta weight, gestational age, and birth weight-to-gestational age ratio) and logistic regression (for pre-eclampsia, preterm birth, LGA, SGA, and GDM). All analyses incorporated matching weights with robust standard error estimation. Effects were stratified by pre-existing diabetes status to account for different treatment indications. For the linear regression, we report the adjusted unit increase by exposure, and for the logistic regression, the adjusted odds ratio (aOR). We also estimated the effect in women with pre-existing diabetes and women without pre-existing diabetes to take into account the two different indications. The resulting estimates of the Average Treatment Effect on the Treated (ATT) are presented with the 95% confidence interval (95% CI). In a sensitivity analysis, we re-estimated the propensity score-weighted models with additional adjustment for maternal pre-pregnancy BMI, maternal age and pre-existing diabetes.

Missing data were imputed using Multiple Imputation by Chained Equations (MICE) with predictive mean matching, running 100 iterations with 10 independent imputations. We used both exposure, confounders, and outcome variables for the imputation. We inspected trace plots and strip plots to verify that the imputation algorithm had converged (Supplementary Figs S14–S17).

All statistical analyses were conducted using R (R Core Team, 2021), version 4.2.3 with packages: MICE (v 3.15.0) for imputations (van Buuren and Groothuis-Oudshoorn, 2011), MatchThem (v 4.5.2) (Ho *et al.*, 2011; Sävje *et al.*, 2021) for propensity score matching, and marginal effects (v0.18.0) for ATT including robust standard errors. Results are reported according to STROBE guidelines.

## Results

### Study characteristics and descriptive data

#### Participant characteristics

From 756 636 eligible pregnancies, we identified 529 pregnancies with periconceptional GLP-1 RA exposure (Fig. 1, Supplementary Fig. S1). Exposed women were older with a higher mean BMI of 34 for women exposed to liraglutide compared to 23 for unexposed women, and a mean BMI of 33 for women exposed to semaglutide compared to 24 for unexposed women (equal to 47.8–37.5% higher BMI, for liraglutide and semaglutide exposure, respectively). Women exposed also had a higher frequency of pre-existing diabetes: 34% compared to 1.5%, and 47% compared to 1.6%, for women exposed to liraglutide or semaglutide, respectively (equaling a 22.7–29.4-fold higher frequency for liraglutide and semaglutide exposure, respectively). A higher number of women exposed to semaglutide were smoking: 17% compared to 9.5% of unexposed women (a 1.8-fold higher frequency) (Table 1).

**Table 1.** Descriptive data on women with a GLP-1 RA exposure in early pregnancy.

Characteristic	**Liraglutide**			**Semaglutide**		
	Unexposed (N = 756 107^1^)	Exposed (N = 295^1^)	*P* value^2^	Unexposed (N = 310 825^1^)	Exposed (N = 234^1^)	*P* value^2^
**Women—N**	479 716	284		248 563	231	
**Maternal age—years**	31.0 (28.0, 34.0)	33.0 (29.0, 37.0)	<0.001	31.0 (28.0, 34.0)	32.0 (29.0, 36.0)	<0.001
**Body mass index** ^†^						
Mean	23.0 (21.0, 27.0)	34.0 (31.0, 38.0)	<0.001	24.0 (21.0, 27.0)	33.0 (30.0, 38.0)	<0.001
Grouped			<0.001			<0.001
<18.5	29 960 (4.1%)	0 (0%)		11 218 (3.7%)	0 (0%)	
18.5–24.9	413 535 (56%)	9 (3.1%)		165 087 (54%)	15 (6.4%)	
25.0–29.9	183 394 (25%)	43 (15%)		79 292 (26%)	40 (17%)	
>30	110 766 (15%)	237 (82%)		49 539 (16%)	178 (76%)	
**Education**			<0.001			<0.001
None or primary	124 194 (16%)	65 (22%)		47 951 (15%)	65 (28%)	
Secondary	282 231 (37%)	143 (48%)		111 188 (36%)	89 (38%)	
Undergraduate (BSc)	210 161 (28%)	65 (22%)		87 837 (28%)	64 (27%)	
Postgraduate (MSc/PhD)	139 521 (18%)	22 (7.5%)		63 849 (21%)	16 (6.8%)	
**Geographic parish in Denmark**			0.001			0.013
Capital Region	276 434 (37%)	105 (36%)		114 862 (37%)	80 (34%)	
Central Region	177 098 (23%)	67 (23%)		73 483 (24%)	52 (22%)	
North Region	71 823 (9.5%)	22 (7.5%)		29 433 (9.5%)	17 (7.3%)	
Region Zealand	85 464 (11%)	56 (19%)		34 813 (11%)	43 (18%)	
Region of Southern Denmark	145 288 (19%)	45 (15%)		58 234 (19%)	42 (18%)	
**Parity**			0.016			0.018
0	339 980 (45%)	135 (46%)		140 269 (45%)	90 (38%)	
1	288 873 (38%)	105 (36%)		121 073 (39%)	90 (38%)	
2	97 305 (13%)	33 (11%)		38 598 (12%)	41 (18%)	
3+	29 949 (4.0%)	22 (7.5%)		10 885 (3.5%)	13 (5.6%)	
**Smoking (yes)**	77 004 (10%)	36 (12%)	0.3	29 351 (9.5%)	40 (17%)	<0.001
**Pre-existing diabetes** ^‡^	11 458 (1.5%)	101 (34%)	<0.001	4996 (1.6%)	110 (47%)	<0.001

N = number of pregnancies.

1 Median (interquartile range, IQR); n (%).

2 Wilcoxon rank sum test; Pearson’s chi-squared test; Fisher’s exact test.

† BMI, the body-mass index is the weight in kilograms divided by the square of the height in meters.

‡ Pre-existing diabetes, excluding gestational diabetes.

Prescriptions of liraglutide and semaglutide stratified by indication (with pre-existing DM or without pre-existing DM) and by year, are shown in Supplementary Table S2. The prescriptions of both liraglutide and semaglutide increased greatly in 2021 and 2022/2023, respectively, especially for women without pre-existing diabetes.

### Outcome data and main results

#### Obstetric complications in the GLP-1 RA-exposed vs. unexposed pregnancies

Women with periconceptional GLP-1 RA exposure had a significantly higher prevalence of pre-eclampsia, GDM, preterm birth, and babies being born large for gestational age (LGA) (Table 2). Results were consistent for both drugs, but not as pronounced for GDM prevalence in pregnancy exposed to semaglutide. Furthermore, placental weight was on average 40–50 g higher, and the gestational age at birth was 8–12 days shorter, with semaglutide exposure associated with the lowest placental weight and the shortest gestational age difference (Table 2). There was no difference in birth weight, but the birth weight: gestational age percentile was higher when exposed to liraglutide or semaglutide (Table 2). No differences were found in stillbirth, placental abruption, or placenta previa (data not shown for privacy reasons).

**Table 2.** Obstetrical outcomes when exposed to liraglutide or semaglutide in early pregnancy.

	**Liraglutide**			Semaglutide		
	Unexposed (N = 756 107^1^)	Exposed (N = 295^1^)	*P* value^2^	Unexposed (N = 310 825^1^)	Exposed (N = 234^1^)	*P-*value^2^
**Pre-eclampsia**	26 361 (3.5%)	27 (9.2%)	<0.001	11 958 (3.8%)	24 (10%)	<0.001
**Gestational diabetes mellitus**	32 217 (4.3%)	29 (9.8%)	<0.001	17 000 (5.5%)	19 (8.1%)	0.075
**Preterm delivery**	37 753 (5.0%)	35 (12%)	<0.001	15 429 (5.0%)	32 (14%)	<0.001
**Gestational age at birth—days**	281 (273, 287)	273 (265, 282)	<0.001	281 (273, 287)	269 (262, 278)	<0.001
**Birth weight child—kg**	3535 (3200, 3866)	3645 (3200, 3960)	0.053	3550 (3212, 3880)	3622 (3231, 3934)	0.13
**Birth weight/gestational age percentile** *	0.50 (0.25, 0.75)	0.62 (0.29, 0.85)	<0.001	0.50 (0.25, 0.75)	0.68 (0.40, 0.87)	<0.001
**LGA** ^†^	75 596 (10.0%)	52 (18%)	<0.001	31 076 (10%)	49 (21%)	<0.001
**SGA** ^‡^	75 600 (10.0%)	28 (9.5%)	0.8	31 076 (10.0%)	26 (11%)	0.60
**Birth weight <2500 g**	26 862 (3.6%)	10 (3.4%)	0.9	10 726 (3.5%)	13 (5.6%)	0.079
**Placental weight—kg**	650 (560, 750)	700 (590, 807)	<0.001	650 (558, 748)	690 (568, 800)	<0.001
**Male child**	388 137 (51%)	151 (51%)	>0.9	150 904 (49%)	118 (50%)	0.60

1 Median (interquartile range, IQR); n (%).

2 Wilcoxon rank sum test; Pearson’s chi-squared test.

* Birth weight/gestational age percentile according to birth year and child sex.

† LGA, large-for-gestational-age, was defined as being in the highest 90th percentile when compared within groups of the same calendar year, the same sex, and the same gestational age category.

‡ SGA, small-for-gestational-age, was defined as being in the lowest 10th percentile when compared within groups of the same calendar year, the same sex, and the same gestational age category.

#### GLP-1 RA-exposed pregnancies compared to matched control groups

**Figure 2.**
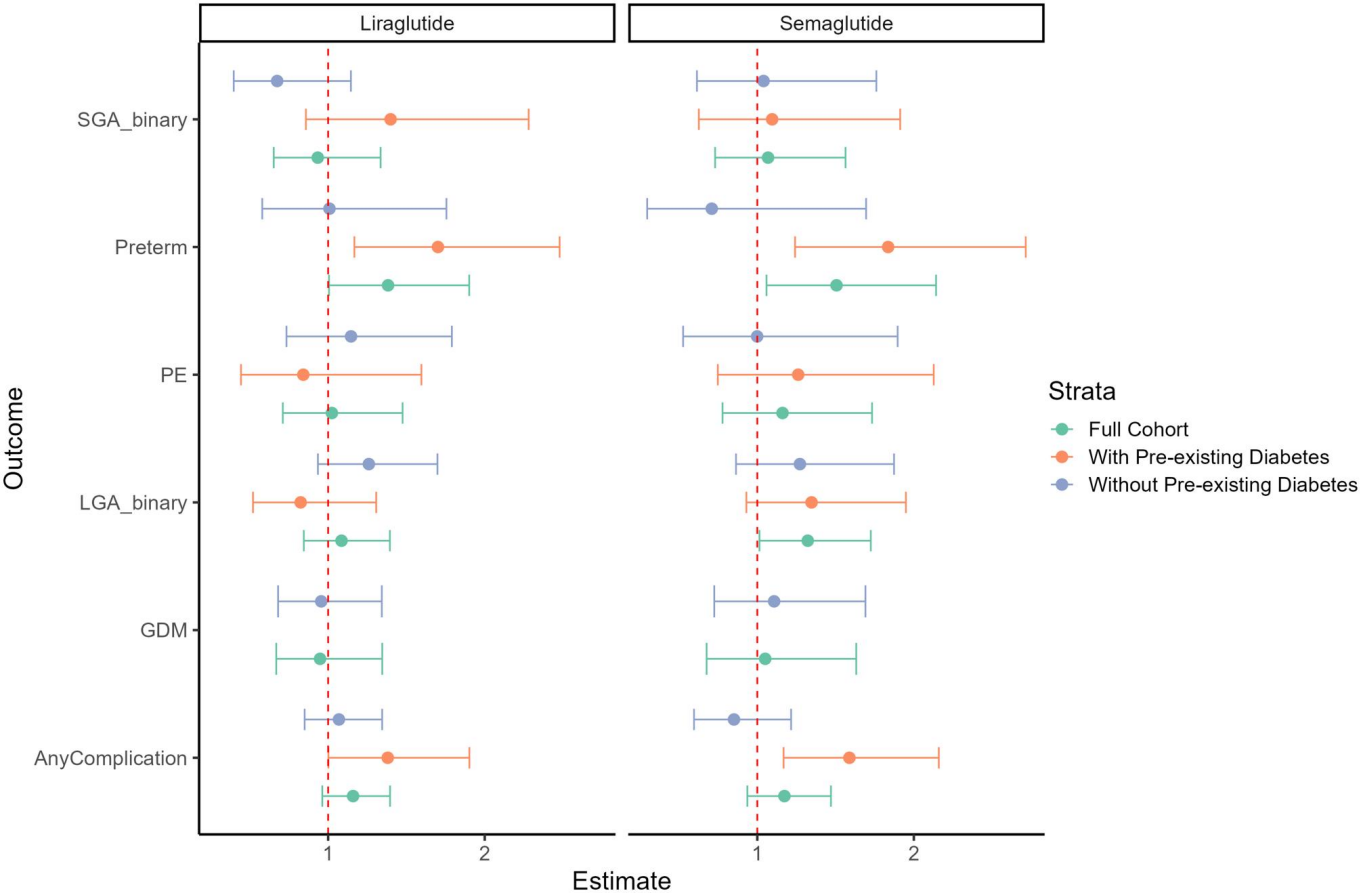
**Associations between periconceptional exposure and obstetric complications**. Relative risk and 95% confidence intervals for the associations between periconceptional exposure to liraglutide or semaglutide and obstetrical complications. Estimates are stratified by pre-existing diabetes status. In the full cohort, no significant change in risk of obstetrical complications was observed with exposure to either GLP1-RA, except for preterm birth. Stratification by pre-existing diabetes indicates that this increased preterm birth risk is primarily observed in the subgroup using GLP1-RAs for weight management, which generally showed elevated risks across obstetrical complications. SGA, small for gestational age; preterm, preterm birth; PE, pre-eclampsia; LGA, large for gestational age; GDM, gestational diabetes mellitus; Any complication: a diagnosis of either pre-eclampsia, gestational diabetes, preterm birth, LGA, or SGA.

When estimating the Average Treatment Effect on the Treated (ATT), we did not observe any differences in birth weight, placental weight or the frequency of SGA or LGA, GDM or pre-eclampsia between GLP-1 RA exposure and any of the six control groups (Fig. 2, Table 3). However, the risk of preterm birth was significantly higher in women periconceptionally exposed to liraglutide (adjusted Odds Ratio, aOR, 1.38, 95% CI 1.01–1.90) and semaglutide (aOR 1.51, 95% CI 1.06–2.14) (Fig. 2, Table 3). The magnitude of the effect increased substantially when the analyses were not adjusted for BMI and pre-existing diabetes, with elevated risks for both liraglutide (aOR 2.07, 95% CI 1.52–2.83) and semaglutide (aOR 2.39, 95% CI 1.72–3.31, Table 3).

**Table 3.** Association between liraglutide or semaglutide exposure and obstetrical outcomes when using balanced covariates.

Outcome	Exposure	Group 1 (all confounders)	Group2 (confounders except BMI and pre-existing diabetes)	Group 3 (5% higher counterfactual BMI)	Group 4 (7.5% higher counterfactual BMI)	Group 5 (10% higher counterfactual BMI)	Group 6 (15% higher counterfactual BMI)
**Pre-eclampsia** ^1^	Liraglutide	1.02 (0.71; 1.48)	2.15 (1.50; 3.09)	0.93 (0.65; 1.34)	0.91 (0.63; 1.32)	0.88 (0.61; 1.27)	0.83 (0.57; 1.20)
	Semaglutide	1.16 (0.78; 1.73)	2.40 (1.64; 3.53)	1.07 (0.71; 1.60)	1.02 (0.68; 1.54)	0.99 (0.65; 1.51)	1.02 (0.66; 1.57)
**Gestational diabetes** ^1^	Liraglutide	0.95 (0.67; 1.35)	1.50 (1.06; 2.13)	0.90 (0.64; 1.28)	0.87 (0.62; 1.24)	0.86 (0.61; 1.23)	0.82 (0.57; 1.17)
	Semaglutide	1.05 (0.68; 1.63)	1.09 (0.71; 1.69)	1.00 (0.64; 1.55)	0.97 (0.62; 1.50)	0.95 (0.61; 1.48)	0.88 (0.56; 1.38)
**Preterm delivery** ^1^	Liraglutide	1.38 (1.01; 1.90)	2.07 (1.52; 2.83)	1.35 (0.98; 1.85)	1.34 (0.98; 1.85)	1.32 (0.96; 1.83)	1.34 (0.97; 1.86)
	Semaglutide	1.51 (1.06; 2.14)	2.39 (1.72; 3.31)	1.51 (1.05; 2.16)	1.51 (1.04; 2.18)	1.56 (1.08; 2.27)	1.55 (1.02; 2.35)
**Gestational age at birth—days** ^2^	Liraglutide	−2.09 (−3.60; −0.58)	−5.41 (−6.90; −3.92)	−1.79 (−3.31; −0.27)	−1.74 (−3.27; −0.20)	−1.57 (−3.12; −0.03)	−1.37 (−2.93; 0.20)
	Semaglutide	−5.70 (−8.25; −3.16)	−9.43 (−11.90; −6.96)	−5.47 (−8.09; −2.85)	−5.51 (−8.08; −2.94)	−5.45 (−8.04; −2.86)	−5.22 (−7.96; −2.49)
**Birth weight child** ^2^ **—kg**	Liraglutide	19.10 (−48.42; 86.62)	74.25 (7.55; 140.96)	17.12 (−50.57; 84.81)	17.49 (−50.66; 85.64)	12.83 (−56.35; 82.01)	10.17 (−59.43; 79.77)
	Semaglutide	−2.69 (−95.82; 90.44)	44.99 (−44.97; 134.95)	−1.65 (−96.61; 93.30)	−12.77 (−108.13; 82.58)	−27.33 (−123.91; 69.24)	−36.89 (−138.51; 64.73)
**Birth weight/**	Liraglutide	0.19 (−0.03; 0.42)	0.54 (0.31; 0.77)	0.18 (−0.05; 0.40)	0.17 (−0.06; 0.40)	0.15 (−0.08; 0.38)	0.13 (−0.10; 0.37)
**Gestational age—percentile** ^2^ *	Semaglutide	0.21 (−0.10; 0.53)	0.56 (0.26; 0.86)	0.21 (−0.11; 0.53)	0.17 (−0.15; 0.49)	0.11 (−0.21; 0.44)	0.07 (−0.27; 0.41)
**LGA** ^1^ **—yes** ^†^	Liraglutide	1.09 (0.84; 1.39)	1.81 (1.41; 2.32)	1.05 (0.82; 1.35)	1.05 (0.82; 1.35)	1.03 (0.80; 1.32)	1.01 (0.78; 1.30)
	Semaglutide	1.32 (1.01; 1.72)	2.11 (1.64; 2.72)	1.29 (0.98; 1.68)	1.26 (0.96; 1.66)	1.19 (0.89; 1.58)	1.11 (0.82; 1.51)
**SGA** ^1^ **—yes** ^‡^	Liraglutide	0.93 (0.65; 1.34)	0.84 (0.59; 1.20)	0.93 (0.65; 1.33)	0.92 (0.64; 1.32)	0.93 (0.65; 1.33)	0.94 (0.66; 1.36)
	Semaglutide	1.07 (0.73; 1.56)	0.98 (0.68; 1.41)	1.10 (0.74; 1.62)	1.09 (0.73; 1.62)	1.09 (0.73; 1.63)	1.10 (0.72; 1.70)
**Placental weight** ^2^ **—kg**	Liraglutide	12.05 (−8.44; 32.55)	48.01 (27.73; 68.29)	8.88 (−11.60; 29.35)	7.56 (−12.91; 28.03)	5.48 (−15.18; 26.13)	2.66 (−18.13; 23.46)
	Semaglutide	1.19 (−22.79; 25.18)	39.29 (16.32; 62.26)	−2.22 (−26.64; 22.20)	−5.64 (−30.05; 18.77)	−11.95 (−37.64; 13.73)	−13.37 (−39.03; 12.29)
**Any obstetric complications** ^1^ ^§^	Liraglutide	1.16 (0.96; 1.40)	1.89 (1.57; 2.27)	1.10 (0.92; 1.33)	1.08 (0.90; 1.30)	1.06 (0.88; 1.28)	1.03 (0.85; 1.25)
	Semaglutide	1.17 (0.94; 1.47)	1.69 (1.37; 2.10)	1.13 (0.90; 1.41)	1.09 (0.87; 1.37)	1.09 (0.87; 1.38)	1.07 (0.83; 1.38)

1 Relative risk and 95% confidence intervals.

2 Linear regression coefficient and 95% confidence interval.

* Birth weight/gestational age percentile according to birth year and child sex.

† LGA, large-for-gestational-age, was defined as being in the highest 90th percentile when compared within groups of the same calendar year, the same sex, and the same gestational age category.

‡ SGA, small-for-gestational-age, was defined as being in the lowest 10th percentile when compared within groups of the same calendar year, the same sex, and the same gestational age category.

§ Pre-eclampsia, gestational diabetes, preterm birth, LGA, SGA.

BMI, body mass index.

**Figure 3.**
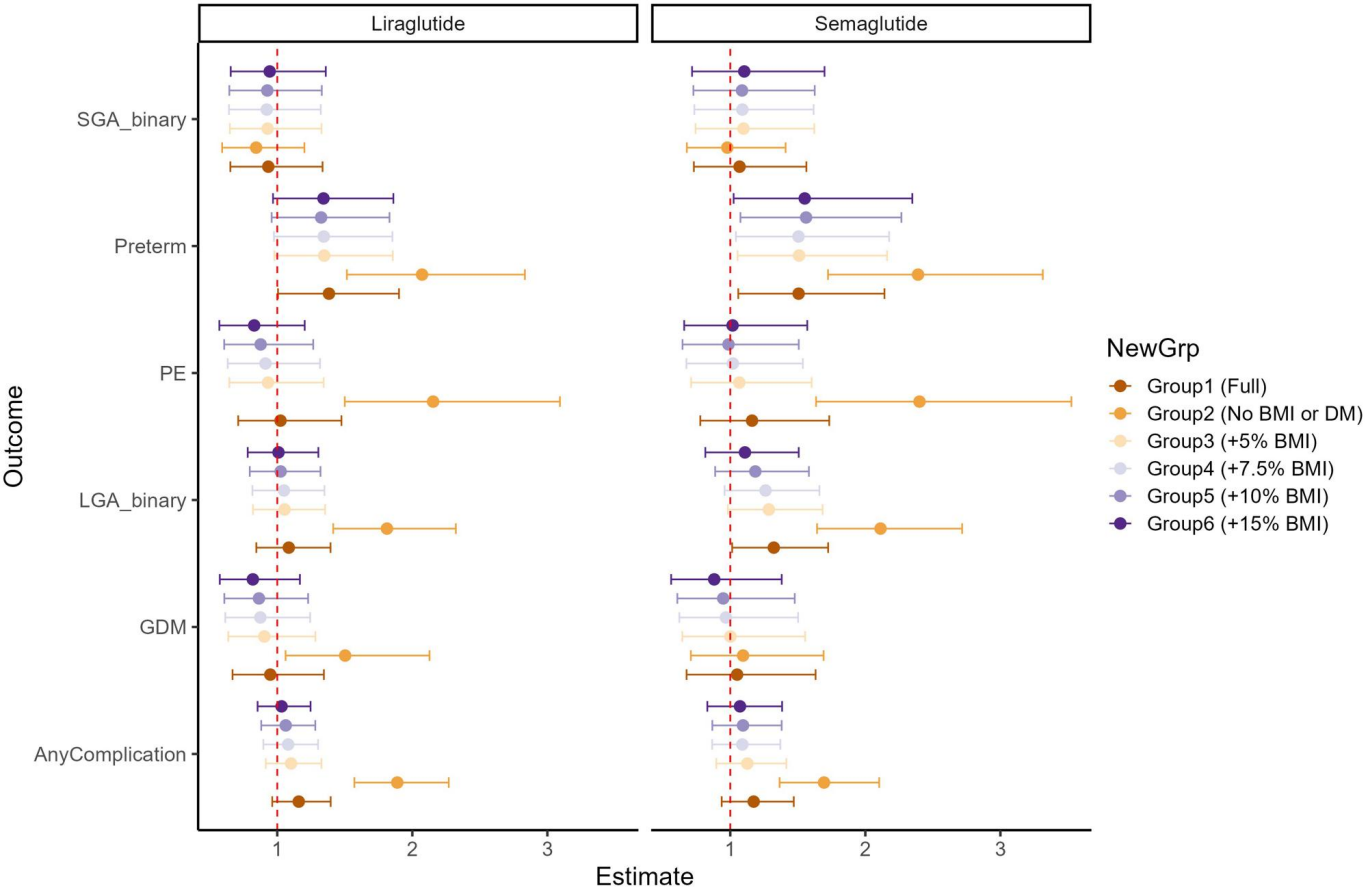
**Sensitivity analyses with counterfactual scenarios for associations between periconceptional exposure and obstetric complications**. Relative risk and 95% confidence intervals for associations between periconceptional exposure to liraglutide or semaglutide and obstetrical complications, with sensitivity analyses using counterfactual scenarios. Estimates from the primary matched analysis are compared with estimates obtained after replacing pre-pregnancy BMI with counterfactual values representing a scenario without GLP-1 RA–related weight reduction. The effect estimates for obstetrical complications were essentially unchanged. However, when pre-existing diabetes and BMI (control group 2) were omitted from the propensity score model, substantially stronger associations between GLP1-RA exposure and obstetrical complications were observed. Grp, group; SGA, small for gestational age; preterm, preterm birth; PE, pre-eclampsia; LGA, large for gestational age; GDM, gestational diabetes mellitus; Any complication: a diagnosis of either pre-eclampsia, gestational diabetes, preterm birth, LGA, or SGA; BMI, body mass index; DM, diabetes mellitus (pre-existing).

In analyses stratified by pre-existing diabetes, we found that the increased risk of preterm birth was only present when GLP-1 RAs were used for diabetes treatment: liraglutide (n = 101, aOR 1.70, 95% CI 1.17–2.48) and semaglutide (n = 110, aOR 1.84, 95% CI 1.24–2.71) (Fig. 2, Tables 4 and 5). Importantly, when used exclusively for weight management, GLP-1 RAs showed no association with preterm birth (liraglutide, n = 194: aOR 1.01, 95% CI 0.58–1.76; semaglutide: n = 124, aOR 0.71, 95% CI 0.30–1.70) (Tables 4 and 5). To assess whether the findings could be explained by GLP-1 RA-related weight loss, we conducted a counterfactual analysis in which we set pre-pregnancy BMI to values consistent with a scenario without treatment-related weight reduction and repeated the analyses. However, this did not alter the main findings (Fig. 3 and Tables 4 and 5). In a sensitivity analysis with additional adjustment for pre-pregnancy BMI, maternal age, and pre-existing diabetes, results were materially unchanged (Supplementary Fig. S18).

**Table 4.** Liraglutide exposure and obstetrical outcomes.

	Group 1 (all confounders)	Group 2 (confounders except BMI and pre-existing diabetes)	Group 3 (5% higher counterfactual BMI)	Group 4 (7.5% higher counterfactual BMI)	Group 5 (10% higher counterfactual BMI)	Group 6 (15% higher counterfactual BMI)
Without pre-existing DM, n = 194						
With pre-existing DM, n = 101						
**Pre-eclampsia** ^1^						
Without pre-existing DM	1.15 (0.73; 1.79)	2.25 (1.44; 3.50)	1.07 (0.68; 1.67)	1.01 (0.65; 1.59)	0.99 (0.63; 1.55)	0.93 (0.59; 1.47)
With pre-existing DM	0.84 (0.44; 1.60)	0.88 (0.46; 1.67)	0.74 (0.39; 1.41)	0.76 (0.40; 1.44)	0.71 (0.37; 1.36)	0.68 (0.35; 1.31)
**Gestational diabetes** ^1^						
Without pre-existing DM	0.96 (0.68; 1.34)	2.24 (1.60; 3.14)	0.91 (0.65; 1.27)	0.88 (0.62; 1.24)	0.86 (0.61; 1.22)	0.82 (0.58; 1.15)
With pre-existing DM	6.96 (5.69; 8.52)	1.37 (1.12; 1.67)	6.73 (5.50; 8.24)	6.77 (5.53; 8.30)	6.73 (5.48; 8.27)	6.56 (5.33; 8.06)
**Preterm delivery** ^1^						
Without pre-existing DM	1.01 (0.58; 1.76)	1.12 (0.64; 1.94)	0.99 (0.56; 1.72)	0.98 (0.56; 1.71)	0.97 (0.56; 1.70)	0.94 (0.54; 1.65)
With pre-existing DM	1.70 (1.17; 2.48)	1.55 (1.07; 2.26)	1.66 (1.14; 2.43)	1.66 (1.14; 2.43)	1.64 (1.11; 2.41)	1.72 (1.17; 2.55)
**Gestational age at birth—days** ^2^						
Without pre-existing DM	−0.39 (−2.10; 1.32)	−1.85 (−3.55; −0.15)	−0.11 (−1.82; 1.61)	0.02 (−1.70; 1.75)	0.11 (−1.62; 1.83)	0.48 (−1.27; 2.22)
With pre-existing DM	−5.27 (−7.67; −2.87)	−4.65 (−7.05; −2.25)	−5.00 (−7.46; −2.53)	−5.08 (−7.53; −2.62)	−4.80 (−7.33; −2.26)	−4.93 (−7.48; −2.37)
**Birth weight child** ^2^ **—kg**						
Without pre-existing DM	68.72 (−10.43; 147.88)	138.27 (59.52; 217.02)	61.60 (−17.84; 141.05)	63.92 (−15.75; 143.59)	59.33 (−20.64; 139.30)	58.54 (−22.14; 139.23)
With pre-existing DM	−75.56 (−198.51; 47.40)	−30.79 (−153.53; 91.95)	−68.01 (−191.28; 55.27)	−71.31 (−195.75; 53.14)	−76.55 (−204.30; 51.21)	−82.98 (−211.46; 45.51)
**Birth weight/gestational age percentile** ^2^ *						
Without pre-existing DM	0.28 (0.03; 0.54)	0.60 (0.34; 0.86)	0.24 (−0.02; 0.50)	0.25 (−0.01; 0.51)	0.23 (−0.04; 0.49)	0.21 (−0.06; 0.47)
With pre-existing DM	0.02 (−0.43; 0.47)	0.14 (−0.30; 0.59)	0.04 (−0.40; 0.49)	0.03 (−0.42; 0.48)	0.00 (−0.46; 0.46)	−0.02 (−0.48; 0.45)
**LGA** ^1^ **—yes** ^†^						
Without pre-existing DM	1.26 (0.93; 1.70)	1.94 (1.44; 2.61)	1.20 (0.89; 1.61)	1.19 (0.88; 1.61)	1.18 (0.88; 1.60)	1.15 (0.85; 1.56)
With pre-existing DM	0.82 (0.52; 1.31)	0.93 (0.58; 1.47)	0.83 (0.52; 1.32)	0.83 (0.52; 1.31)	0.79 (0.49; 1.26)	0.79 (0.49; 1.27)
**SGA** ^1^—**yes**^‡^						
Without pre-existing DM	0.67 (0.40; 1.15)	0.60 (0.35; 1.01)	0.68 (0.40; 1.15)	0.67 (0.39; 1.14)	0.68 (0.40; 1.16)	0.67 (0.39; 1.15)
With pre-existing DM	1.40 (0.86; 2.28)	1.25 (0.77; 2.02)	1.37 (0.84; 2.23)	1.38 (0.84; 2.25)	1.35 (0.82; 2.22)	1.46 (0.88; 2.41)
**Placental weight** ^2^ **—kg**						
Without pre-existing DM	28.24 (3.57; 52.91)	55.91 (31.38; 80.44)	23.58 (−1.15; 48.31)	22.95 (−1.68; 47.58)	20.67 (−3.99; 45.33)	17.90 (−7.24; 43.05)
With pre-existing DM	−19.43 (−56.12; 17.25)	1.63 (−35.08; 38.34)	−19.49 (−56.13; 17.16)	−22.17 (−59.02; 14.68)	−23.68 (−61.51; 14.14)	−26.52 (−64.01; 10.98)
**Any obstetric complications** ^1^ ^§^						
Without pre-existing DM	1.07 (0.85; 1.34)	1.88 (1.50; 2.37)	1.02 (0.81; 1.28)	0.99 (0.79; 1.25)	0.98 (0.78; 1.23)	0.93 (0.74; 1.18)
With pre-existing DM	1.38 (1.00; 1.90)	1.36 (0.99; 1.87)	1.30 (0.94; 1.79)	1.31 (0.95; 1.80)	1.26 (0.91; 1.76)	1.28 (0.92; 1.79)

Regression coefficients and odds ratios across outcomes, for the full cohort and stratified by pre-existing diabetes mellitus.

1 Relative risk and 95% confidence intervals.

2 Linear regression coefficient and 95% confidence interval.

* Birth weight/gestational age percentile according to birth year and child sex.

† LGA, large-for-gestational-age, was defined as being in the highest 90th percentile when compared within groups of the same calendar year, the same sex, and the same gestational age category.

‡ SGA, small-for-gestational-age, was defined as being in the lowest 10th percentile when compared within groups of the same calendar year, the same sex, and the same gestational age category.

§ Pre-eclampsia, gestational diabetes, preterm birth, LGA, SGA.

DM, diabetes mellitus.

BMI, body mass index.

**Table 5.** Semaglutide exposure and obstetrical outcomes.

	Group 1 (all confounders)	Group 2 (confounders except BMI and pre-existing diabetes)	Group 3 (5% higher counterfactual BMI)	Group 4 (7.5% higher counterfactual BMI)	Group 5 (10% higher counterfactual BMI)	Group 6 (15% higher counterfactual BMI)
Without pre-existing DM, n = 124						
With pre-existing DM, n = 110						
**Pre-eclampsia** ^1^						
Without pre-existing DM	1.00 (0.53; 1.90)	1.75 (0.93; 3.31)	0.93 (0.49; 1.76)	0.88 (0.46; 1.67)	0.83 (0.44; 1.58)	0.79 (0.42; 1.52)
With pre-existing DM	1.26 (0.75; 2.13)	1.30 (0.77; 2.18)	1.15 (0.67; 1.97)	1.11 (0.64; 1.92)	1.10 (0.61; 1.96)	1.22 (0.66; 2.24)
**Gestational diabetes** ^1^						
Without pre-existing DM	1.11 (0.73; 1.69)	2.02 (1.33; 3.08)	1.05 (0.69; 1.60)	1.01 (0.66; 1.55)	0.99 (0.65; 1.51)	0.92 (0.60; 1.41)
With pre-existing DM	7.43 (6.06; 9.11)	1.47 (1.20; 1.80)	7.31 (5.93; 9.01)	7.16 (5.79; 8.86)	7.06 (5.67; 8.79)	6.91 (5.43; 8.79)
**Preterm delivery** ^1^						
Without pre-existing DM	0.71 (0.30; 1.70)	0.73 (0.31; 1.74)	0.69 (0.29; 1.64)	0.69 (0.29; 1.65)	0.66 (0.27; 1.58)	0.68 (0.28; 1.64)
With pre-existing DM	1.84 (1.24; 2.71)	1.55 (1.04; 2.30)	1.88 (1.25; 2.83)	1.87 (1.23; 2.85)	2.05 (1.32; 3.18)	1.99 (1.18; 3.35)
**Gestational age at birth—days** ^2^						
Without pre-existing DM	−2.09 (−4.38; 0.20)	−3.02 (−5.29; −0.75)	−1.84 (−4.14; 0.45)	−1.77 (−4.06; 0.53)	−1.53 (−3.83; 0.77)	−1.44 (−3.76; 0.89)
With pre-existing DM	−9.37 (−13.78; −4.96)	−8.93 (−13.34; −4.53)	−9.21 (−13.81; −4.61)	−9.38 (−13.84; −4.91)	−9.59 (−14.09; −5.09)	−9.21 (−14.10; −4.32)
**Birth weight child** ^2^ **—kg**						
Without pre-existing DM	52.99 (−45.16; 151.13)	112.03 (14.65; 209.41)	−8.93 (−13.34; −4.53)	37.17 (−61.55; 135.88)	35.76 (−64.99; 136.51)	20.92 (−79.28; 121.12)
With pre-existing DM	−63.75 (−228.68; 101.18)	12.06 (−151.79; 175.91)	−48.90 (−219.63; 121.83)	−66.87 (−238.36; 104.63)	−97.95 (−270.44; 74.55)	−101.03 (−288.16; 86.10)
**Birth weight/Gestational age percentile** ^2^ *						
Without pre-existing DM	0.30 (−0.02; 0.63)	0.56 (0.24; 0.89)	0.26 (−0.07; 0.58)	0.23 (−0.10; 0.56)	0.22 (−0.12; 0.55)	0.16 (−0.18; 0.49)
With pre-existing DM	0.10 (−0.45; 0.66)	0.36 (−0.19; 0.91)	0.15 (−0.42; 0.72)	0.09 (−0.49; 0.67)	−0.01 (−0.59; 0.57)	−0.04 (−0.66; 0.59)
**LGA** ^1^ **—yes** ^†^						
Without pre-existing DM	1.27 (0.86; 1.87)	1.81 (1.24; 2.66)	1.20 (0.81; 1.76)	1.16 (0.79; 1.71)	1.14 (0.77; 1.69)	1.07 (0.72; 1.60)
With pre-existing DM	1.35 (0.93; 1.95)	1.57 (1.09; 2.26)	1.36 (0.92; 1.99)	1.35 (0.90; 2.01)	1.22 (0.79; 1.86)	1.13 (0.70; 1.81)
**SGA** ^1^ **—yes** ^‡^						
Without pre-existing DM	1.04 (0.61; 1.76)	0.93 (0.55; 1.56)	1.09 (0.64; 1.84)	1.09 (0.65; 1.85)	1.07 (0.62; 1.82)	1.10 (0.65; 1.87)
With pre-existing DM	1.09 (0.63; 1.91)	0.89 (0.50; 1.57)	1.10 (0.62; 1.98)	1.08 (0.59; 1.97)	1.11 (0.60; 2.05)	1.11 (0.55; 2.22)
**Placental weight** ^2^ **—kg**						
Without pre-existing DM	13.44 (−15.34; 42.21)	39.45 (10.87; 68.02)	8.24 (−20.53; 37.01)	5.78 (−23.15; 34.71)	2.40 (−26.71; 31.50)	−0.39 (−29.59; 28.81)
With pre-existing DM	−14.38 (−54.15; 25.39)	6.01 (−32.82; 44.83)	−15.34 (−57.23; 26.56)	−19.98 (−61.38; 21.42)	−29.69 (−73.69; 14.30)	−29.36 (−74.30; 15.58)
**Any obstetric complications** ^1^ ^§^						
Without pre-existing DM	0.85 (0.60; 1.22)	1.30 (0.91; 1.86)	0.81 (0.57; 1.15)	0.78 (0.55; 1.12)	0.75 (0.53; 1.08)	0.72 (0.50; 1.03)
With pre-existing DM	1.59 (1.17; 2.16)	1.45 (1.07; 1.97)	1.55 (1.13; 2.13)	1.49 (1.08; 2.07)	1.59 (1.13; 2.24)	1.63 (1.06; 2.50)

Regression coefficients and odds ratios across outcomes, for the full cohort and stratified by pre-existing diabetes mellitus.

1 Relative risk and 95% confidence intervals.

2 Linear regression coefficient and 95% confidence interval.

* Birth weight/gestational age percentile according to birth year and child sex.

† LGA, large-for-gestational-age, was defined as being in the highest 90th percentile when compared within groups of the same calendar year, the same sex, and the same gestational age category.

‡ SGA, small-for-gestational-age, was defined as being in the lowest 10th percentile when compared within groups of the same calendar year, the same sex, and the same gestational age category.

§ Pre-eclampsia, gestational diabetes, preterm birth, LGA, SGA.

DM, diabetes mellitus.

BMI, body mass index.

## Discussion

In this nationwide study conducted in Denmark, we show that periconceptional GLP-1 RA exposure was associated with a higher risk of adverse obstetrical complications, pre-eclampsia, GDM, preterm birth, and for babies to be born LGA, among 756 636 pregnancies with 529 exposed pregnancies. After adjusting for measured confounders using a propensity score matching method, we only found evidence for an increased risk of preterm birth. This increased risk was only observed in women with pre-existing diabetes, and not those using GLP-1 RAs for weight management. This suggests that GLP-1 RA exposure itself may not be driving the increased risk, but rather factors related to the underlying indication for prescription (i.e., diabetes) may be contributory. We additionally performed counterfactual experiments with assumed weight loss, but this did not alter our findings.

### Interpretation

Obesity and diabetes increase pregnancy complications. Therefore, optimizing obesity and diabetes treatment in reproductive-age women is important to reduce the risk of obstetrical complications and also increase the likelihood of natural conception (Nohr *et al.*, 2008; Best *et al.*, 2017). Established programs and recommendations for when to screen and monitor women with obesity, diabetes, or incident GDM already exist, but action prior to pregnancy or periconceptional would be ideal (Tsakiridis *et al.*, 2021). Besides lifestyle interventions, bariatric surgery is an alternative intervention for weight loss that might reduce weight; however, this method increases the risk of maternal malnutrition and obstetric complications, e.g. SGA and preterm birth. GLP-1 RA-based therapy has introduced a new era in the treatment of overweight and obesity, which is reflected in numerous clinical trials and patient-reported outcomes, highlighting its efficacy and safety profile (Kushner *et al.*, 2020; Davies *et al.*, 2021; Rubino *et al.*, 2021; Wadden *et al.*, 2021; Wilding *et al.*, 2021; Garvey *et al.*, 2022; Weghuber *et al.*, 2022; Lincoff *et al.*, 2023; Wilkinson *et al.*, 2023). However, no pharmacological weight loss treatments are approved periconceptionally or in pregnancy (Nuako *et al.*, 2023). The prescription of GLP-1 RAs has increased markedly within the last 4–8 years as an anti-diabetic drug and increased additionally following the approval of semaglutide for weight loss treatment (Cesta *et al.*, 2024; Møller and Hegelund, 2024). In Denmark, as 70% of users of weight loss medication are women, it would be inevitable that inadvertent periconceptional GLP-1 RA exposure would occur (Ladebo *et al.*, 2024). Thus, continuous investigation of periconceptional health effects is warranted.

Recent studies on GLP-1 RA safety in early pregnancy remain limited (Muller *et al.*, 2023; Cesta *et al.*, 2024; Dao *et al.*, 2024; Maslin *et al.*, 2024; Drummond *et al.*, 2025; Finkle and Brost, 2025; Parker *et al.*, 2025; Price and Nankervis, 2025). One study presented cases of obstetric complications without statistical analyses, but found comparable rates of pregnancy loss, a decreased rate of preterm births, and an increased number of elective terminations following exposure to a GLP-1 RA (Dao *et al.*, 2024). Two studies (Dao *et al.* and Cesta *et al.*) reported no elevated risk of major congenital malformations, which the few published case reports also have shown (Greco, 2015; Ivanišević *et al.*, 2018; Muller *et al.*, 2023; Skov *et al.*, 2023; Cesta *et al.*, 2024; Dao *et al.*, 2024), and the study by Hanif *et al.* (2025) reported no increased risk of fetal cardiac or fetal kidney anomalies. Parker *et al.* (2025) showed similar results when examining data from RCTs from the US FDA. Animal model organism studies have, however, indicated a reduction in fetal growth (European Medicines Agency, 2019; European Medicines Agency, 2022a; European Medicines Agency, 2022b; European Medicines Agency, 2024). Yet, we did not observe any differences in birth weight in our present study. In the first study by Cesta *et al.*, the risk in patients with pregestational type 2 diabetes was examined, comparing the risk to patients treated with insulin during pregnancy (Cesta *et al.*, 2024). The second study by Dao *et al.* compared pregnancy outcomes in women exposed to GLP1-RAs in early pregnancy (n = 168), combining all GLP1-RA drugs despite the differences in effectiveness, either for diabetes or obesity treatment with those in two reference groups: (i) women with diabetes exposed to at least one non-GLP1-RA anti-diabetic drug during the first trimester and (ii) a reference group of overweight/obese women without diabetes, between 2009 and 2022 (Dao *et al.*, 2024). No difference in major congenital malformations or increased risk of pregnancy losses between the three groups was found in the crude or adjusted analyses. Although patients with type 2 diabetes are a patient group in which the risk of major congenital malformations is higher than the general population, these studies present the first results on the risk of major congenital malformation after periconceptional GLP-1 RA exposure in women (Dolk *et al.*, 2010; Ornoy *et al.*, 2015). However, the potential risk of obstetric complications specifically for semaglutide, the next generation of GLP-1 RA, has not been previously evaluated.

### Generalizability

This study provides potentially important evidence for counselling women inadvertently exposed to GLP1-RAs. We found no increased risk of obstetric complications, except for preterm birth when used for diabetes treatment. Preterm birth is a well-known complication of pregnancy for women with pre-existing diabetes (Jensen *et al.*, 2004; Ludvigsson *et al.*, 2019). The risk increases further with poor glycemic control for both medically induced and spontaneous preterm birth (Ludvigsson *et al.*, 2019). Our findings demonstrate the critical need for indication-specific research on GLP-1 RA exposure during early pregnancy to establish a comprehensive safety profile. Such research could determine whether or not GLP-1 RAs can be safely used periconceptionally for weight management to enhance natural conception rates and reduce pregnancy complications, ultimately improving both maternal and offspring health outcomes. Further evidence in prospective studies and randomized control trials of the safety of GLP-1 RAs during pregnancy is urgently needed before a potential recommendation can be made and its benefits can be used for future patients and mothers-to-be.

### Limitations and strengths

This study is based on comprehensive longitudinal data from multiple nationwide registries in Denmark, where information can be linked across registries due to the unique personal identification number. The GLP-1 RA exposure is based on the redemption of a prescription of liraglutide or semaglutide. It is mandatory, by law, to report the redemption of prescriptions and births to the national registry, which provides the study of both a long and a complete follow-up period of every pregnancy. A very large study population facilitated proper weighting and matching using propensity scoring, in which we accounted for the two primary indications for GLP1-RA treatment: diabetes and obesity, yielding balanced estimates of the effect on obstetric outcomes. Furthermore, we could control socioeconomic factors, including geographic region and education. However, residual confounding is always a limitation to the observational design. We did not perform specific analyses excluding women with a history of bariatric surgery, and we cannot exclude that this may have influenced the results for a small subgroup. Another limitation of the study is the lack of knowledge about GLP-1 RA compliance post prescription redemption; however, compliance is considered to be high given the high cost of the drug and the wish for weight loss. We also cannot rule out that there may be an increased trend amongst patients receiving GLP-1 RAs as a treatment of diabetes to be more likely to continue its use during the period of preconception, although this hypothesis needs independent confirmation. Misclassification of unexposed also has to be taken into account due to possible parallel import of GLP-1 RA and thus not registered as a prescription in the national registry. The primary and approved use of GLP-1 RAs in the study time period was diabetes, and weight loss treatment was off-label treatment for most of the study time. Semaglutide for weight loss treatment was first approved and marketed in Denmark from December 2022. However, the use of GLP-1 RAs for weight loss treatment increased rapidly, and 65% and 53% of our included exposed pregnancies were under the indication weight loss (of liraglutide and semaglutide exposure, respectively). The lack of association between GLP-1 RAs and weight loss for preterm birth could be due to power issues, therefore, further studies are needed. We also explored different models for propensity score matching that yielded highly concordant results. Lastly, when excluding BMI and pre-existing diabetes from the propensity score model, we observed similar findings to completely unadjusted estimates, for both semaglutide and liraglutide. It is also important to note that the observational design cannot establish causality.

## Conclusion

Our findings suggest that diabetes itself, rather than preconceptional GLP-1 RA exposure, may be the likely cause of the association with risk of preterm birth. This should be explicitly accounted for in future studies.

## Supplementary Material

hoag015_Supplementary_Data

## Data Availability

According to Danish law, scientific organizations can be authorized to work with data within Statistics Denmark and can provide access to individual scientists inside and outside of Denmark. Data are available via the Research Service Department at Statistics Denmark: www.dst.dk/da/TilSalg/Forskningsservice for researchers who meet the criteria for access to confidential data.
