# Supplementary material for: Periconceptional GLP-1 receptor agonist exposure and obstetric outcomes: a Danish nationwide cohort study
Source: Hum Reprod Open. 2026 Mar 18;2026(2):hoag015. doi: 10.1093/hropen/hoag015 (PMC12995393; doi:10.1093/hropen/hoag015)
Supplement: hoag015_Supplementary_Data [file hoag015_supplementary_data.zip › HRO-25-0325-R3-Supplementary Data_EO.docx]

**Supplementary File S1: Covariate balance statistics across six confounder-adjustment methods (as defined in Table 3A-B), including polynomial terms up to degree 2 and all two-way interactions** (provided as separate file)**.**

**Supplementary Table S1: ICD-10 codes for the outcome diagnosis**

| **Diagnosis** | **ICD-10 codes** |
| --- | --- |
| Pre-eclampsia | DO140 |
|  | DO141 |
|  | DO149 |
| Gestational diabetes mellitus | DO244 |
|  | DO244B |
|  | DO244C |
|  | DO244D |
|  | DO244E |
| Placental abruption | DO450 |
|  | DP458 |
|  | DP459 |
| Placental previa | DO440 |
|  | DO440A |
|  | DO440A |
|  | DO441 |
|  | DO441A |
|  | DO441B |
|  | DO449 |

ICD-10 codes: the International Statistical Classification of Diseases and Related Health Problems, Tenth Revision

**Supplementary Table S2: Shows the numbers of Liraglutide and Semaglutide prescriptions stratified by indication (with pre-existing DM or without pre-existing DM) and by year**

| Year of prescription | Liraglutide * | | Semaglutide † | |
| --- | --- | --- | --- | --- |
|  | With out pre-existing DM | With pre-existing DM | With out pre-existing DM | With pre-existing DM |
| 2012 | 12 | 9 | 0 | 0 |
| 2013 | 16 | 13 | 0 | 0 |
| 2014 | 9 | 7 | 0 | 0 |
| 2015 | 9 | 5 | 0 | 0 |
| 2016 | 11 | 8 | 0 | 0 |
| 2017 | 15 | 9 | 0 | 0 |
| 2018 | 19 | 12 | 0 | 0 |
| 2019 | 21 | 10 | 0 | 0 |
| 2020 | 15 | 6 | 18 | 17 |
| 2021 | 39 | 9 | 28 | 21 |
| 2022 | 43 | 6 | 40 | 22 |
| 2023 | 93 | 7 | 155 | 50 |

* Data before 2012 is merged due to small numbers and privacy of patients

† Data before 2020 is merged due to small numbers and privacy of patients

DM - diabetes mellitus

**
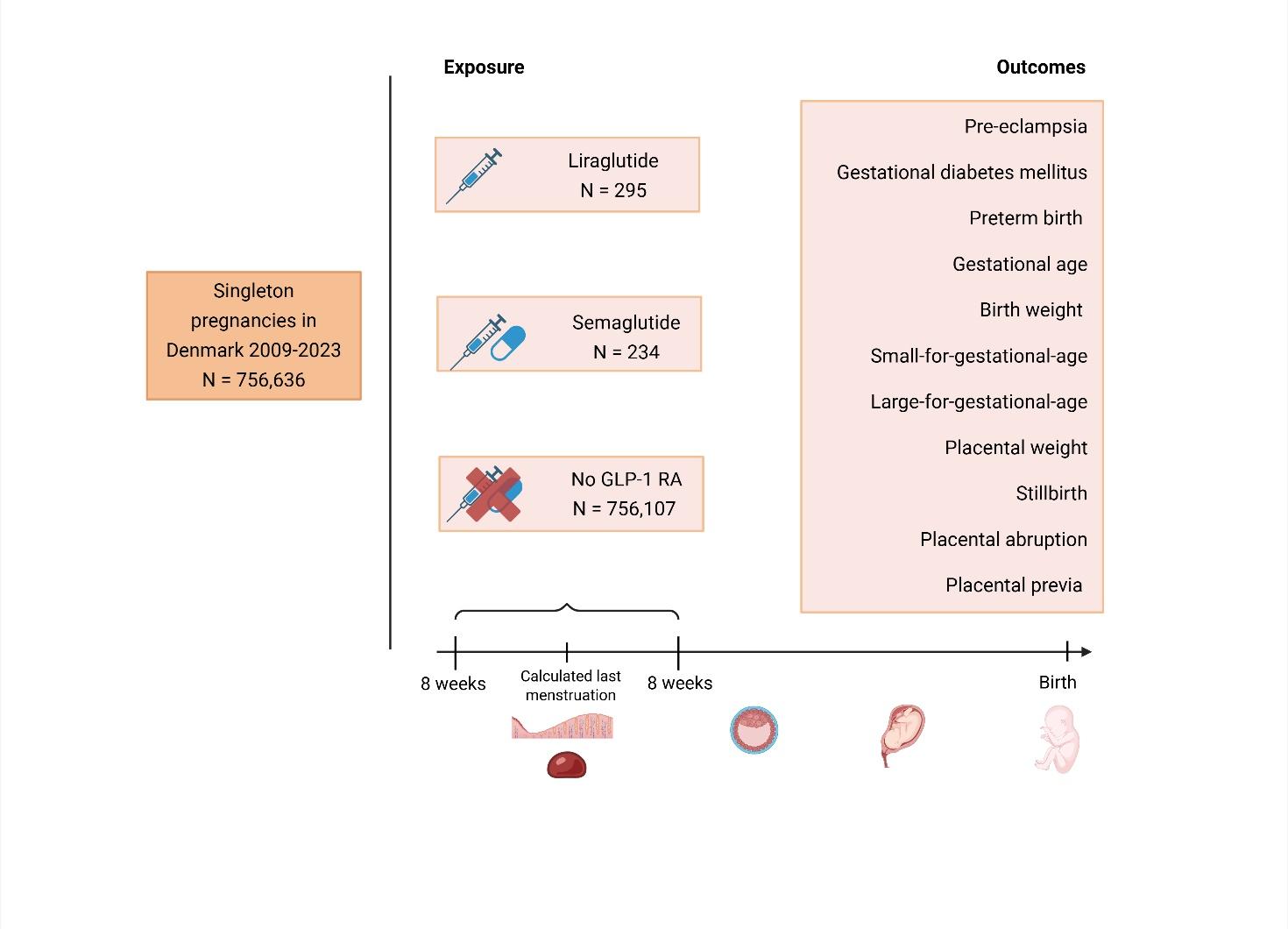
**

**Supplementary Figure S1:**

**Study Overview. From 756,636 eligible pregnancies, we identified 529 pregnancies with periconceptional GLP-1 RA exposure.**

**Supplementary Figure S2: Love plots for the matching for Liraglutide, Group 1**. This figure displays the standardized mean differences (SMDs) for the pre-specified covariates before and after propensity score matching. The plot includes the SMD and variance ratio for each covariate. The vertical reference lines at an SMD of 0.1 indicate the threshold for acceptable balance. Covariates are deemed balanced if their absolute SMD is below this threshold in the matched sample (blue dots). A Variance Ratio close to 1.0 indicates that the matching/weighting procedure was successful in equating the spread (variance) of the covariate distributions. The vertical reference lines at 0.5 and 2.0 represent the accepted range for adequate variance balance.

Education level: UDD_CAT_1NoneOrPrimaryEducation - no formal/primary, UDD_CAT_2SecondaryEducation – secondary, UDD_CAT_3Undergraduate – undergraduate, UDD_CAT_4Postgraduate - postgraduate.

Geographic parish of residence: Region_Region_Hovedstaden - Capital Region, Region_Region _Midtjylland - Central Region, Region_Region _Nordjylland - North Region, Region_Region_Sjælland - Region Zealand, Region_Region _Syddanmark - Region of Southern Denmark.

Year_numeric - year of pregnancy

Month_numeric - month of pregnancy

PreDM - pre-existing diabetes mellitus

BMI - body mass index

**Supplementary Figure S3: Love plots for the matching for Liraglutide, Group 2.** This figure displays the standardized mean differences (SMDs) for the pre-specified covariates before and after propensity score matching. The plot includes the SMD and variance ratio for each covariate. The vertical reference lines at an SMD of 0.1 indicate the threshold for acceptable balance. Covariates are deemed balanced if their absolute SMD is below this threshold in the matched sample (blue dots). A Variance Ratio close to 1.0 indicates that the matching/weighting procedure was successful in equating the spread (variance) of the covariate distributions. The vertical reference lines at 0.5 and 2.0 represent the accepted range for adequate variance balance.

Education level: UDD_CAT_1NoneOrPrimaryEducation - no formal/primary, UDD_CAT_2SecondaryEducation – secondary, UDD_CAT_3Undergraduate – undergraduate, UDD_CAT_4Postgraduate - postgraduate.

Geographic parish of residence: Region_Region_Hovedstaden - Capital Region, Region_Region _Midtjylland - Central Region, Region_Region _Nordjylland - North Region, Region_Region_Sjælland - Region Zealand, Region_Region _Syddanmark - Region of Southern Denmark.

Year_numeric - year of pregnancy

Month_numeric - month of pregnancy

PreDM - pre-existing diabetes mellitus

BMI - body mass index

**Supplementary Figure S4: Love plots for the matching for Liraglutide, Group 3.** This figure displays the standardized mean differences (SMDs) for the pre-specified covariates before and after propensity score matching. The plot includes the SMD and variance ratio for each covariate. The vertical reference lines at an SMD of 0.1 indicate the threshold for acceptable balance. Covariates are deemed balanced if their absolute SMD is below this threshold in the matched sample (blue dots). A Variance Ratio close to 1.0 indicates that the matching/weighting procedure was successful in equating the spread (variance) of the covariate distributions. The vertical reference lines at 0.5 and 2.0 represent the accepted range for adequate variance balance.

Education level: UDD_CAT_1NoneOrPrimaryEducation - no formal/primary, UDD_CAT_2SecondaryEducation – secondary, UDD_CAT_3Undergraduate – undergraduate, UDD_CAT_4Postgraduate - postgraduate.

Geographic parish of residence: Region_Region_Hovedstaden - Capital Region, Region_Region _Midtjylland - Central Region, Region_Region _Nordjylland - North Region, Region_Region_Sjælland - Region Zealand, Region_Region _Syddanmark - Region of Southern Denmark.

Year_numeric - year of pregnancy

Month_numeric - month of pregnancy

PreDM - pre-existing diabetes mellitus

BMI - body mass index

**Supplementary Figure S5: Love plots for the matching for Liraglutide, Group 4.** This figure displays the standardized mean differences (SMDs) for the pre-specified covariates before and after propensity score matching. The plot includes the SMD and variance ratio for each covariate. The vertical reference lines at an SMD of 0.1 indicate the threshold for acceptable balance. Covariates are deemed balanced if their absolute SMD is below this threshold in the matched sample (blue dots). A Variance Ratio close to 1.0 indicates that the matching/weighting procedure was successful in equating the spread (variance) of the covariate distributions. The vertical reference lines at 0.5 and 2.0 represent the accepted range for adequate variance balance.

Education level: UDD_CAT_1NoneOrPrimaryEducation - no formal/primary, UDD_CAT_2SecondaryEducation – secondary, UDD_CAT_3Undergraduate – undergraduate, UDD_CAT_4Postgraduate - postgraduate.

Geographic parish of residence: Region_Region_Hovedstaden - Capital Region, Region_Region _Midtjylland - Central Region, Region_Region _Nordjylland - North Region, Region_Region_Sjælland - Region Zealand, Region_Region _Syddanmark - Region of Southern Denmark.

Year_numeric - year of pregnancy

Month_numeric - month of pregnancy

PreDM - pre-existing diabetes mellitus

BMI - body mass index

**Supplementary Figure S6: Love plots for the matching for Liraglutide, Group 5.** This figure displays the standardized mean differences (SMDs) for the pre-specified covariates before and after propensity score matching. The plot includes the SMD and variance ratio for each covariate. The vertical reference lines at an SMD of 0.1 indicate the threshold for acceptable balance. Covariates are deemed balanced if their absolute SMD is below this threshold in the matched sample (blue dots). A Variance Ratio close to 1.0 indicates that the matching/weighting procedure was successful in equating the spread (variance) of the covariate distributions. The vertical reference lines at 0.5 and 2.0 represent the accepted range for adequate variance balance.

Education level: UDD_CAT_1NoneOrPrimaryEducation - no formal/primary, UDD_CAT_2SecondaryEducation – secondary, UDD_CAT_3Undergraduate – undergraduate, UDD_CAT_4Postgraduate - postgraduate.

Geographic parish of residence: Region_Region_Hovedstaden - Capital Region, Region_Region _Midtjylland - Central Region, Region_Region _Nordjylland - North Region, Region_Region_Sjælland - Region Zealand, Region_Region _Syddanmark - Region of Southern Denmark.

Year_numeric - year of pregnancy

Month_numeric - month of pregnancy

PreDM - pre-existing diabetes mellitus

BMI - body mass index

**Supplementary Figure S7: Love plots for the matching for Liraglutide, Group 6.** This figure displays the standardized mean differences (SMDs) for the pre-specified covariates before and after propensity score matching. The plot includes the SMD and variance ratio for each covariate. The vertical reference lines at an SMD of 0.1 indicate the threshold for acceptable balance. Covariates are deemed balanced if their absolute SMD is below this threshold in the matched sample (blue dots). A Variance Ratio close to 1.0 indicates that the matching/weighting procedure was successful in equating the spread (variance) of the covariate distributions. The vertical reference lines at 0.5 and 2.0 represent the accepted range for adequate variance balance.

Education level: UDD_CAT_1NoneOrPrimaryEducation - no formal/primary, UDD_CAT_2SecondaryEducation – secondary, UDD_CAT_3Undergraduate – undergraduate, UDD_CAT_4Postgraduate - postgraduate.

Geographic parish of residence: Region_Region_Hovedstaden - Capital Region, Region_Region _Midtjylland - Central Region, Region_Region _Nordjylland - North Region, Region_Region_Sjælland - Region Zealand, Region_Region _Syddanmark - Region of Southern Denmark.

Year_numeric - year of pregnancy

Month_numeric - month of pregnancy

PreDM - pre-existing diabetes mellitus

BMI - body mass index

**Supplementary Figure S8: Love plots for the matching for Semaglutide, Group 1.** This figure displays the standardized mean differences (SMDs) for the pre-specified covariates before and after propensity score matching. The plot includes the SMD and variance ratio for each covariate. The vertical reference lines at an SMD of 0.1 indicate the threshold for acceptable balance. Covariates are deemed balanced if their absolute SMD is below this threshold in the matched sample (blue dots). A Variance Ratio close to 1.0 indicates that the matching/weighting procedure was successful in equating the spread (variance) of the covariate distributions. The vertical reference lines at 0.5 and 2.0 represent the accepted range for adequate variance balance.

Education level: UDD_CAT_1NoneOrPrimaryEducation - no formal/primary, UDD_CAT_2SecondaryEducation – secondary, UDD_CAT_3Undergraduate – undergraduate, UDD_CAT_4Postgraduate - postgraduate.

Geographic parish of residence: Region_Region_Hovedstaden - Capital Region, Region_Region _Midtjylland - Central Region, Region_Region _Nordjylland - North Region, Region_Region_Sjælland - Region Zealand, Region_Region _Syddanmark - Region of Southern Denmark.

Year_numeric - year of pregnancy

Month_numeric - month of pregnancy

PreDM - pre-existing diabetes mellitus

BMI - body mass index

**Supplementary Figure S9: Love plots for the matching for Semaglutide, Group 2.** This figure displays the standardized mean differences (SMDs) for the pre-specified covariates before and after propensity score matching. The plot includes the SMD and variance ratio for each covariate. The vertical reference lines at an SMD of 0.1 indicate the threshold for acceptable balance. Covariates are deemed balanced if their absolute SMD is below this threshold in the matched sample (blue dots). A Variance Ratio close to 1.0 indicates that the matching/weighting procedure was successful in equating the spread (variance) of the covariate distributions. The vertical reference lines at 0.5 and 2.0 represent the accepted range for adequate variance balance.

Education level: UDD_CAT_1NoneOrPrimaryEducation - no formal/primary, UDD_CAT_2SecondaryEducation – secondary, UDD_CAT_3Undergraduate – undergraduate, UDD_CAT_4Postgraduate - postgraduate.

Geographic parish of residence: Region_Region_Hovedstaden - Capital Region, Region_Region _Midtjylland - Central Region, Region_Region _Nordjylland - North Region, Region_Region_Sjælland - Region Zealand, Region_Region _Syddanmark - Region of Southern Denmark.

Year_numeric - year of pregnancy

Month_numeric - month of pregnancy

PreDM - pre-existing diabetes mellitus

BMI - body mass index

**Supplementary Figure S10: Love plots for the matching for Semaglutide, Group 3.** This figure displays the standardized mean differences (SMDs) for the pre-specified covariates before and after propensity score matching. The plot includes the SMD and variance ratio for each covariate. The vertical reference lines at an SMD of 0.1 indicate the threshold for acceptable balance. Covariates are deemed balanced if their absolute SMD is below this threshold in the matched sample (blue dots). A Variance Ratio close to 1.0 indicates that the matching/weighting procedure was successful in equating the spread (variance) of the covariate distributions. The vertical reference lines at 0.5 and 2.0 represent the accepted range for adequate variance balance.

Education level: UDD_CAT_1NoneOrPrimaryEducation - no formal/primary, UDD_CAT_2SecondaryEducation – secondary, UDD_CAT_3Undergraduate – undergraduate, UDD_CAT_4Postgraduate - postgraduate.

Geographic parish of residence: Region_Region_Hovedstaden - Capital Region, Region_Region _Midtjylland - Central Region, Region_Region _Nordjylland - North Region, Region_Region_Sjælland - Region Zealand, Region_Region _Syddanmark - Region of Southern Denmark.

Year_numeric - year of pregnancy

Month_numeric - month of pregnancy

PreDM - pre-existing diabetes mellitus

BMI - body mass index

**Supplementary Figure S11: Love plots for the matching for Semaglutide, Group 4.** This figure displays the standardized mean differences (SMDs) for the pre-specified covariates before and after propensity score matching. The plot includes the SMD and variance ratio for each covariate. The vertical reference lines at an SMD of 0.1 indicate the threshold for acceptable balance. Covariates are deemed balanced if their absolute SMD is below this threshold in the matched sample (blue dots). A Variance Ratio close to 1.0 indicates that the matching/weighting procedure was successful in equating the spread (variance) of the covariate distributions. The vertical reference lines at 0.5 and 2.0 represent the accepted range for adequate variance balance.

Education level: UDD_CAT_1NoneOrPrimaryEducation - no formal/primary, UDD_CAT_2SecondaryEducation – secondary, UDD_CAT_3Undergraduate – undergraduate, UDD_CAT_4Postgraduate - postgraduate.

Geographic parish of residence: Region_Region_Hovedstaden - Capital Region, Region_Region _Midtjylland - Central Region, Region_Region _Nordjylland - North Region, Region_Region_Sjælland - Region Zealand, Region_Region _Syddanmark - Region of Southern Denmark.

Year_numeric - year of pregnancy

Month_numeric - month of pregnancy

PreDM - pre-existing diabetes mellitus

BMI - body mass index

**Supplementary Figure S12: Love plots for the matching for Semaglutide, Group 5.** This figure displays the standardized mean differences (SMDs) for the pre-specified covariates before and after propensity score matching. The plot includes the SMD and variance ratio for each covariate. The vertical reference lines at an SMD of 0.1 indicate the threshold for acceptable balance. Covariates are deemed balanced if their absolute SMD is below this threshold in the matched sample (blue dots). A Variance Ratio close to 1.0 indicates that the matching/weighting procedure was successful in equating the spread (variance) of the covariate distributions. The vertical reference lines at 0.5 and 2.0 represent the accepted range for adequate variance balance.

Education level: UDD_CAT_1NoneOrPrimaryEducation - no formal/primary, UDD_CAT_2SecondaryEducation – secondary, UDD_CAT_3Undergraduate – undergraduate, UDD_CAT_4Postgraduate - postgraduate.

Geographic parish of residence: Region_Region_Hovedstaden - Capital Region, Region_Region _Midtjylland - Central Region, Region_Region _Nordjylland - North Region, Region_Region_Sjælland - Region Zealand, Region_Region _Syddanmark - Region of Southern Denmark.

Year_numeric - year of pregnancy

Month_numeric - month of pregnancy

PreDM - pre-existing diabetes mellitus

BMI - body mass index

**Supplementary Figure S13: Love plots for the matching for Semaglutide, Group 6.** This figure displays the standardized mean differences (SMDs) for the pre-specified covariates before and after propensity score matching. The plot includes the SMD and variance ratio for each covariate. The vertical reference lines at an SMD of 0.1 indicate the threshold for acceptable balance. Covariates are deemed balanced if their absolute SMD is below this threshold in the matched sample (blue dots). A Variance Ratio close to 1.0 indicates that the matching/weighting procedure was successful in equating the spread (variance) of the covariate distributions. The vertical reference lines at 0.5 and 2.0 represent the accepted range for adequate variance balance.

Education level: UDD_CAT_1NoneOrPrimaryEducation - no formal/primary, UDD_CAT_2SecondaryEducation – secondary, UDD_CAT_3Undergraduate – undergraduate, UDD_CAT_4Postgraduate - postgraduate.

Geographic parish of residence: Region_Region_Hovedstaden - Capital Region, Region_Region _Midtjylland - Central Region, Region_Region _Nordjylland - North Region, Region_Region_Sjælland - Region Zealand, Region_Region _Syddanmark - Region of Southern Denmark.

Year_numeric - year of pregnancy

Month_numeric - month of pregnancy

PreDM - pre-existing diabetes mellitus

BMI - body mass index


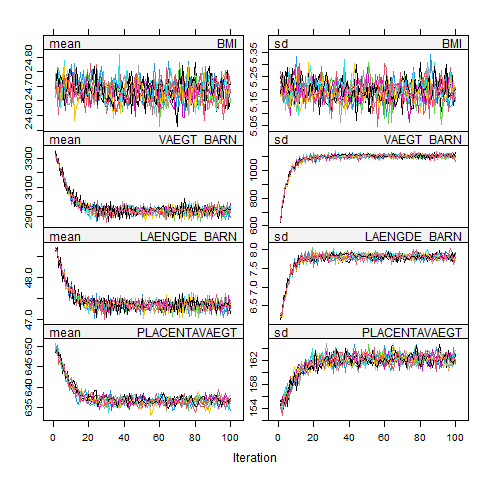


**Supplementary Figure S14: Imputation trace plots used to assess convergence of the multiple imputation model for Liraglutide exposure**.

This figure displays the traceplots for the coefficients of the key variables included in the imputation model used to impute missing data. Each line represents the value of a coefficient across sequential iterations of the imputation Markov Chain Monte Carlo (MCMC) algorithm. Successful convergence is indicated by traces that mix well, appear highly erratic, and show no discernible trends or drift over time, suggesting the chain has reached a stationary distribution. These plots confirm the stability and reliability of the imputed values used in the primary analysis.

BMI - body mass index, Vaegt barn - weight, child, Laengde barn - length, child, Placentavaegt - weight of the placenta


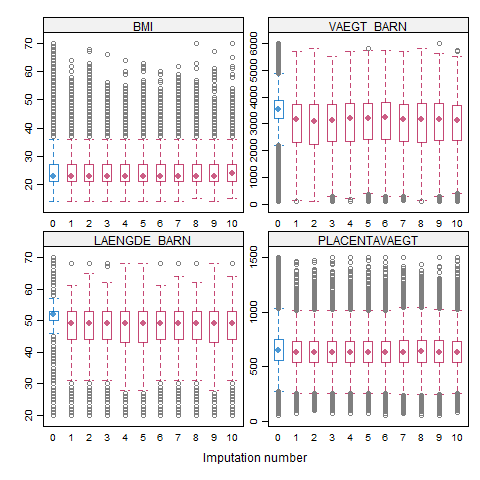


**Supplementary Figure S15: Box-and-whisker plots comparing the distribution of observed versus imputed data for key continuous covariates in the Liraglutide analysis.**

This figure displays the distribution of values (median, quartiles, and range) for selected continuous covariates before (observed data) and after (imputed data) the application of the multiple imputation procedure. The purpose is to visually assess the plausibility of the imputed values. Successful imputation is indicated when the distributions of the imputed data closely resemble the distributions of the observed data, particularly showing similar medians and interquartile ranges, confirming that the imputation process did not introduce systematic distortion into the covariate distributions for the Liraglutide analysis.

BMI - body mass index, Vaegt barn - weight, child, Laengde barn - length, child, Placentavaegt - weight of the placenta


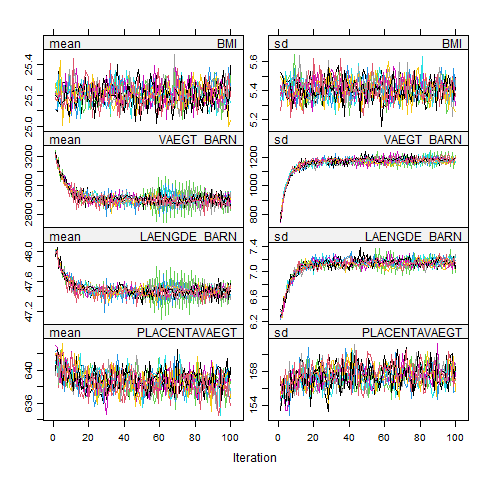


**Supplementary Figure S16: Imputation traceplots used to assess convergence of the multiple imputation model for Semaglutide exposure.**

This figure displays the traceplots for the coefficients of the key variables included in the imputation model used to impute missing data. Each line represents the value of a coefficient across sequential iterations of the imputation Markov Chain Monte Carlo (MCMC) algorithm. Successful convergence is indicated by traces that mix well, appear highly erratic, and show no discernible trends or drift over time, suggesting the chain has reached a stationary distribution. These plots confirm the stability and reliability of the imputed values used in the primary analysis.

BMI - body mass index, Vaegt barn - weight, child, Laengde barn - length, child, Placentavaegt - weight of the placenta

**
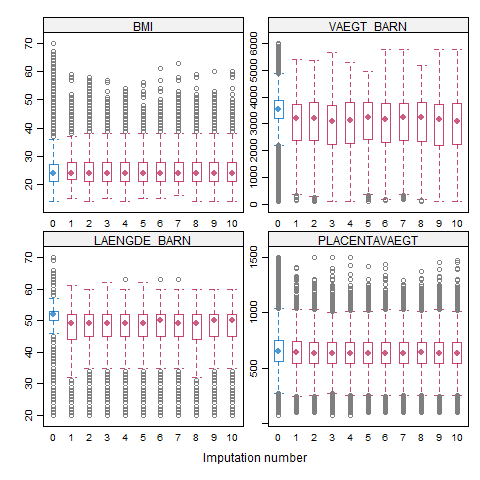
**

**Supplementary Figure S17**: **Box-and-whisker plots comparing the distribution of observed versus imputed data for key continuous covariates in the Semaglutide analysis**.

This figure displays the distribution of values (median, quartiles, and range) for selected continuous covariates before (observed data) and after (imputed data) the application of the multiple imputation procedure. The purpose is to visually assess the plausibility of the imputed values. Successful imputation is indicated when the distributions of the imputed data closely resemble the distributions of the observed data, particularly showing similar medians and interquartile ranges, confirming that the imputation process did not introduce systematic distortion into the covariate distributions for the Semaglutide analysis.

BMI - body mass index, Vaegt barn - weight, child, Laengde barn - length, child, Placentavaegt - weight of the placenta

**
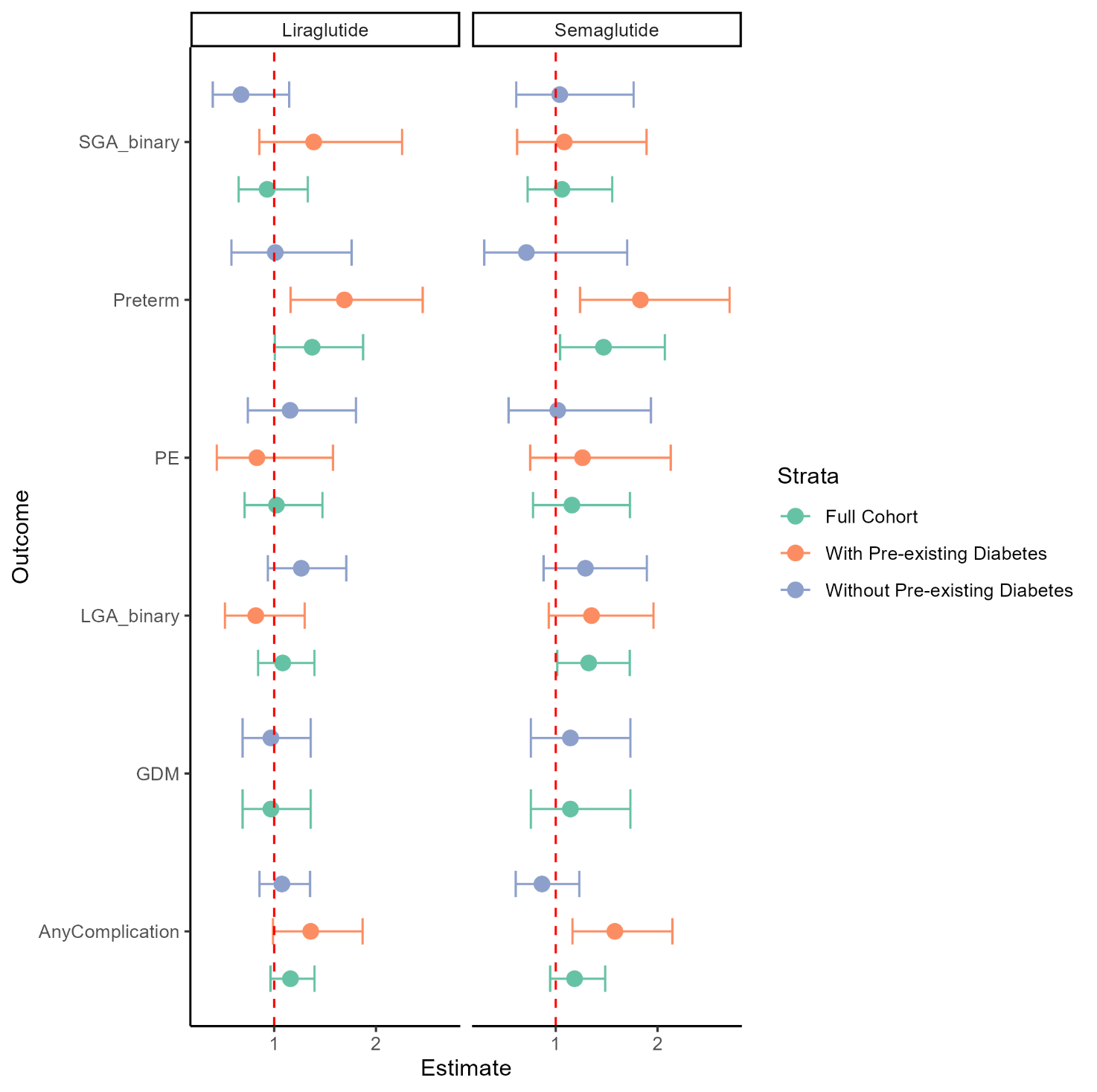
**

**Supplementary Figure S18: Relative risk and 95% confidence intervals for the associations between periconceptional exposure to liraglutide or semaglutide and obstetrical complications.**

Estimates are stratified by pre-existing diabetes status, and additionally adjusted for pre-existing diabetes, BMI, and age. In the full cohort, no significant change in risk of obstetrical complications was observed with exposure to either GLP1-RA, except for preterm birth. Stratification by pre-existing diabetes indicates that this increased preterm birth risk is primarily observed in the subgroup using GLP1-RA for weight management, which generally showed elevated risks across obstetrical complications.

SGA_binary - small for gestational age binary

Preterm - preterm birth

PE - pre-eclampsia

LGA_binary - large for gestational age binary

GDM - gestational diabetes mellitus

AnyComplication - a diagnosis of either pre-eclampsia, gestational diabetes, preterm birth, LGA, or SGA
